# Supplementary material for: Interval breast cancer is associated with other types of tumors
Source: Nat Commun. 2019 Oct 22;10:4648. doi: 10.1038/s41467-019-12652-1 (PMC6805891; doi:10.1038/s41467-019-12652-1)
Supplement: Supplementary file 1 — Supplementary Information [file 41467_2019_12652_MOESM1_ESM.pdf]

Interval breast cancer is associated with other types of tumors

Grassmann et al. 2019 – Supplementary Information

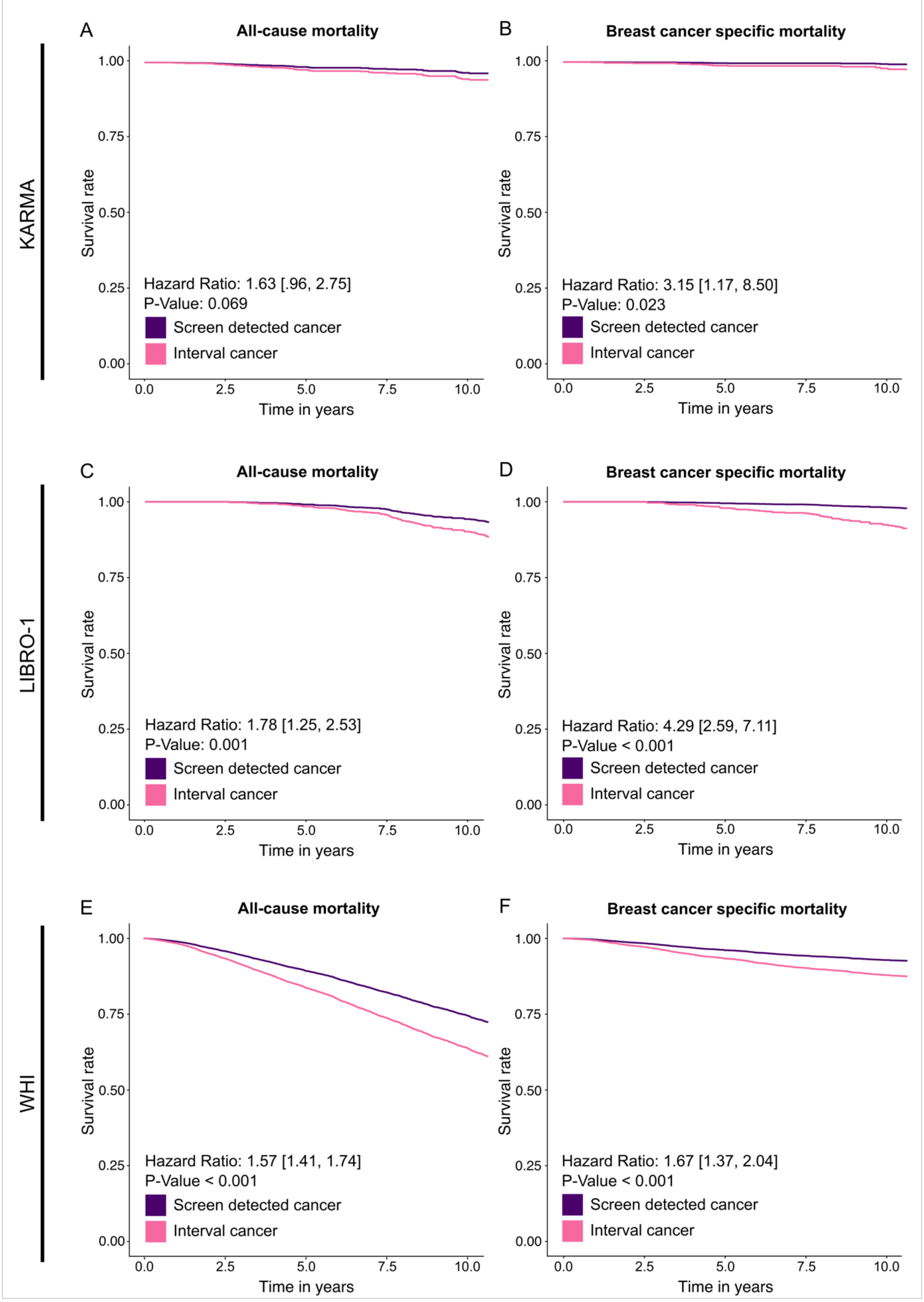

Supplementary Figure 1. All-cause and breast cancer specific mortality by detection mode in three studies. The overall (all-cause) survival (**A, C, E**) and the breast cancer specific survival (**B, D, F**) are shown according to the detection type of the breast cancer. Screen-detected cancers from KARMA (N=1,857), LIBRO-1 (N=1,698) and WHI (N=9,519) generally have a better prognosis compared to interval cancers from KARMA (N=395), LIBRO-1 (N=224) and WHI (N=1,153). The hazard ratio and 95% confidence intervals (in square brackets) were computed with cox proportional hazard models, adjusted for age at diagnosis and, additionally for ethnicity in the WHI.

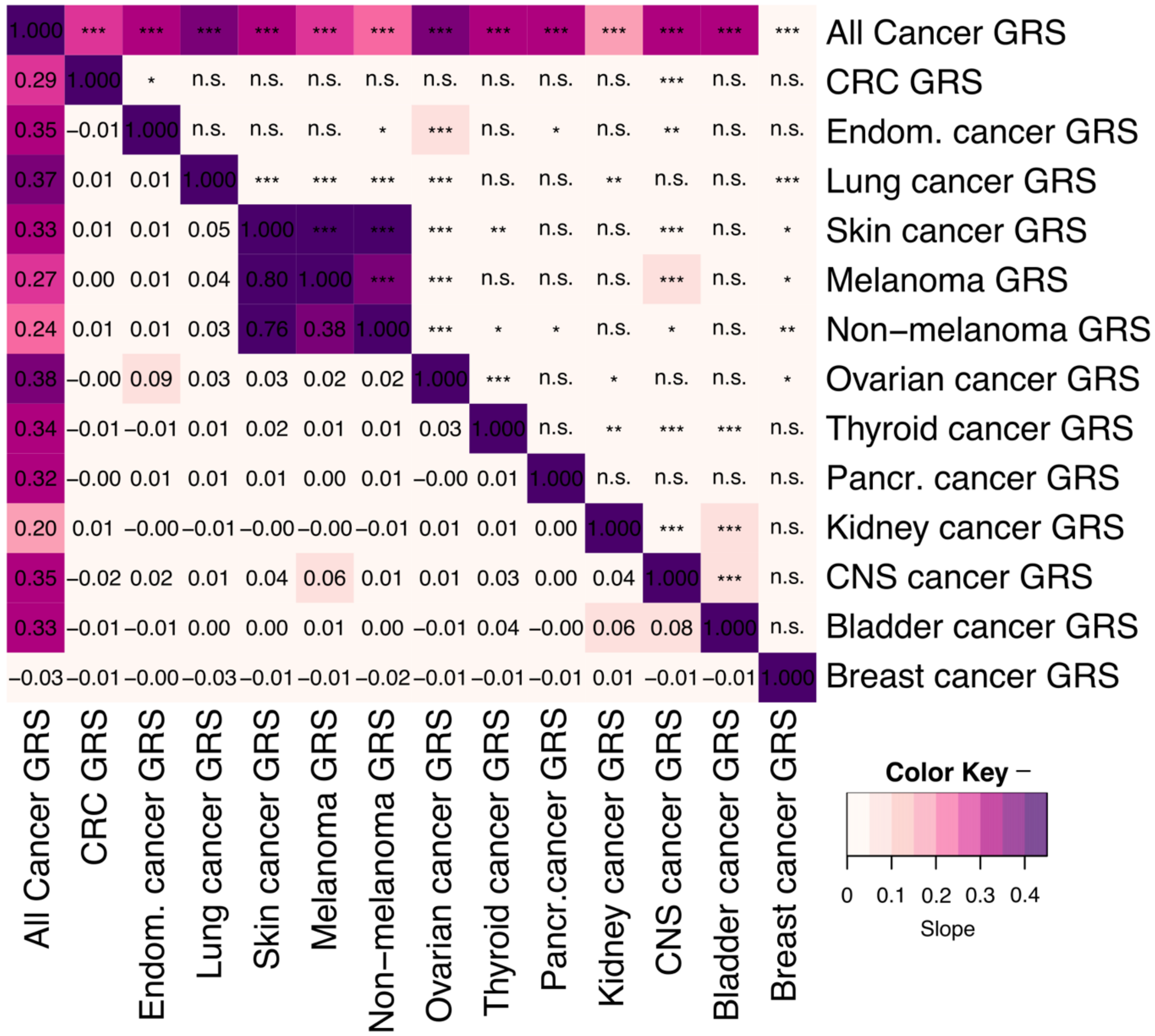

Supplementary Figure 2. Heatmap of regression coefficients between different genetic risk scores. The pairwise correlation between different genetic risk scores (GRS) computed in Caucasian patients from the LIBRO-1 (N=1,784), KARMA (N=1,690) and WHI (N=1,585) studies was investigated with linear regression models, adjusted for the first three principal components of ancestry and study. The slope/regression coefficient is shown on the lower triangle of the heatmap and color coded according to the color key. The statistical significance in the model is indicated by asterisks in the upper triangle. * P < 0.05; ** P < 0.01; ** P < 0.001; n.s: P > 0.05; CRC = colorectal cancer; CNS = central nervous system

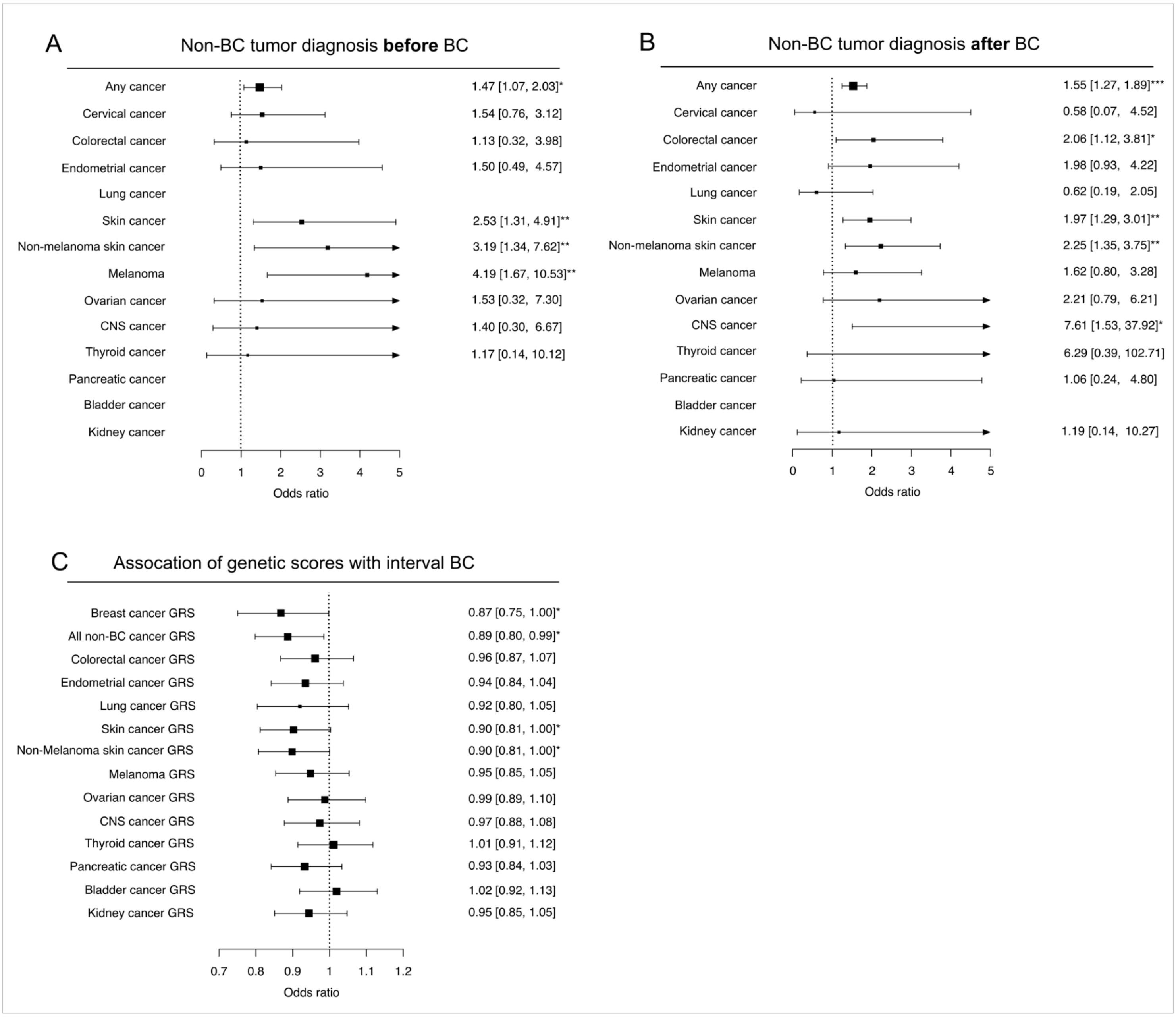

Supplementary Figure 3. Prior and subsequent non-BC tumor diagnoses as well as genetic risk scores in IC compared to SDC, excluding patients from the Women's Health Initiative. The effect size estimates of the association of prior (**A**) as well as subsequent (**B**) non-BC tumor diagnoses on interval breast cancer (IC) risk (black squares) compared to screen-detected breast cancer (SDC) as well as the 95% confidence intervals (CI, horizontal lines) are shown. The estimates were derived from logistic regression models adjusted for age at diagnosis are given on the right-hand side of the plot with the accompanying 95% CI. (**C**) Cancer genetic risk scores (GRS) were computed in Caucasian patients from the LIBRO-1 (N=1,784) and KARMA (N=1,690) studies and the association with interval cancer risk was estimated with logistic regression, adjusted for age at diagnosis, study and the first three principal components. The GRS effect sizes are given per standard deviation of the score. * $P < 0.05$; ** $P < 0.01$; *** $P < 0.001$
