# Supplementary material for: Interval breast cancer is associated with other types of tumors
Source: Nat Commun. 2019 Oct 22;10:4648. doi: 10.1038/s41467-019-12652-1 (PMC6805891; doi:10.1038/s41467-019-12652-1)
Supplement: Supplementary file 2 — Description of Additional Supplementary Files [file 41467_2019_12652_MOESM2_ESM.docx]

Description of Additional Supplementary Files

**Supplementary Data 1. Variants used for the computation of different genetic risk scores.** Chromosomal position coordinates are based on build hg19. We provide the effect size (beta, log odds ratio) of the effect allele (EA) of the variant as well as, where reported, the effect allele frequency in Europeans. TRAIT denotes the trait the association was reported for and SCORE signifies which variant contributed to a specific GRS. We also used logistic regression models, adjusted for age at diagnosis, study and the first three principal components to evaluate the association of the individual variants with interval breast cancer risk in 5059 Caucasian patients (759 IC/4,300 SDC) and report the odds ratio, 95% confidence intervals as well as the P-value of. In addition, we provide the effect allele frequency of individual variants (EAF) in the three cohorts (LIBRO-1, KARMA and WHI). We also flagged variants (INFO field) where we could not unanimously determine the effect (i.e. risk increasing) allele or which were not imputed in at least two out of three datasets.
