# Supplementary material for: Interval breast cancer is associated with other types of tumors
Source: Nat Commun. 2019 Oct 22;10:4648. doi: 10.1038/s41467-019-12652-1 (PMC6805891; doi:10.1038/s41467-019-12652-1)
Supplement: Supplementary file 5 — Reporting Summary [file 41467_2019_12652_MOESM5_ESM.pdf]

nature research

Corresponding author(s): Felix Grassmann
Last updated by author(s): 02-08-2019

Reporting Summary

Nature Research wishes to improve the reproducibility of the work that we publish. This form provides structure for consistency and transparency in reporting. For further information on Nature Research policies, see Authors & Referees and the Editorial Policy Checklist.

Statistics

For all statistical analyses, confirm that the following items are present in the figure legend, table legend, main text, or Methods section.

n/a	Confirmed	
☐	☒	The exact sample size (n) for each experimental group/condition, given as a discrete number and unit of measurement
☐	☒	A statement on whether measurements were taken from distinct samples or whether the same sample was measured repeatedly
☐	☒	The statistical test(s) used AND whether they are one- or two-sided<br>*Only common tests should be described solely by name; describe more complex techniques in the Methods section.*
☐	☒	A description of all covariates tested
☐	☒	A description of any assumptions or corrections, such as tests of normality and adjustment for multiple comparisons
☐	☒	A full description of the statistical parameters including central tendency (e.g. means) or other basic estimates (e.g. regression coefficient) AND variation (e.g. standard deviation) or associated estimates of uncertainty (e.g. confidence intervals)
☐	☒	For null hypothesis testing, the test statistic (e.g. F, t, r) with confidence intervals, effect sizes, degrees of freedom and P value noted<br>*Give P values as exact values whenever suitable.*
☒	☐	For Bayesian analysis, information on the choice of priors and Markov chain Monte Carlo settings
☒	☐	For hierarchical and complex designs, identification of the appropriate level for tests and full reporting of outcomes
☒	☐	Estimates of effect sizes (e.g. Cohen's d, Pearson's r), indicating how they were calculated

Our web collection on statistics for biologists contains articles on many of the points above.

Software and code

Policy information about availability of computer code

Data collection	Data from dbGAP was downloaded with Aspera connect and decrypted with the SRA toolkit provided by the NIH.
Data analysis	Association analyses as well as survival analyses were computed in R using open source packages, as mentioned in the Methods section. The code to compute the phenotypes of the WHI participants is available at https://github.com/FelixGrassmann/IC_WHI

For manuscripts utilizing custom algorithms or software that are central to the research but not yet described in published literature, software must be made available to editors/reviewers. We strongly encourage code deposition in a community repository (e.g. GitHub). See the Nature Research guidelines for submitting code & software for further information.

Data

Policy information about availability of data

All manuscripts must include a data availability statement. This statement should provide the following information, where applicable:

- Accession codes, unique identifiers, or web links for publicly available datasets
- A list of figures that have associated raw data
- A description of any restrictions on data availability

Data from the Women's Health Initiative is available through dbGAP (accession phs000200.v11.p3). Access to phenotypes and biospecimen from the KARMA study can be requested from https://karmastudy.org/data-access/.

Field-specific reporting

Please select the one below that is the best fit for your research. If you are not sure, read the appropriate sections before making your selection.

☒ Life sciences ☐ Behavioural & social sciences ☐ Ecological, evolutionary & environmental sciences

For a reference copy of the document with all sections, see nature.com/documents/nr-reporting-summary-flat.pdf

Life sciences study design

All studies must disclose on these points even when the disclosure is negative.

Sample size	The present study is currently one of the largest studies of interval breast cancer with genetic data. No prior power calculations were performed since no prior studies were available to estimate proper effect size estimates for the present study.
Data exclusions	Samples were excluded based on lack or irregularity of their breast cancer screening history or based on breast features that influence mammographic detection of breast cancer. In the WHI, we excluded breast cancer patients that were diagnosed with breast cancer at autopsy, since this diagnosis date is not reliable enough to determine accurate interval cancer status.
Replication	The study included three independent cohorts with different study design and screening protocols.
Randomization	No randomization was necessary since we compared interval to screen detected breast cancer patients and no treatment was tested.
Blinding	The lead analysts were not involved in recruitment of the samples. The WHI study was recruited completely independent of the authors since the data was accessed via dbGAP.

Reporting for specific materials, systems and methods

We require information from authors about some types of materials, experimental systems and methods used in many studies. Here, indicate whether each material, system or method listed is relevant to your study. If you are not sure if a list item applies to your research, read the appropriate section before selecting a response.

Materials & experimental systems

n/a	Involved in the study	
☒	☐	Antibodies
☒	☐	Eukaryotic cell lines
☒	☐	Palaeontology
☒	☐	Animals and other organisms
☐	☒	Human research participants
☒	☐	Clinical data

Methods

n/a	Involved in the study	
☒	☐	ChIP-seq
☒	☐	Flow cytometry
☒	☐	MRI-based neuroimaging

Human research participants

Policy information about studies involving human research participants

Population characteristics	All participants were at least 45 years of age (average age around 60 at diagnosis in the Swedish cohorts and around 70 in the WHI) and exclusively females with breast cancer. In the Women's Health Initiative, all women were post-menopausal.
Recruitment	Population based recruitment through Swedish registry data. The Women's Health Initiative was a prospective study recruited in the US and accessed via dbGAP
Ethics oversight	Ethical review board at Karolinska Institutet (Stockholm, Sweden); institutional review boards of the institutions participating in the Women's Health Initiative

Note that full information on the approval of the study protocol must also be provided in the manuscript.
